# Combined Nutrition in Very-Low-Birth-Weight Preterm Infants in the Neonatal Intensive Care Unit

**DOI:** 10.7759/cureus.43202

**Published:** 2023-08-09

**Authors:** José Ramón Jiménez- Jiménez, Jose Alfredo Sierra-Ramírez, Rodolfo Rivas-Ruiz, Leonardo Cruz-Reynoso, Marta Elena Hernández-Caballero

**Affiliations:** 1 Neonatal Intensive Care Unit, Hospital de Gineco Obstetricia No. 3, Dr. Víctor Manuel Espinoza de los Reyes Sánchez, Centro Médico Nacional La Raza, Mexico CIty, MEX; 2 Postgraduate Studies and Research Section, Escuela Superior de Medicina, Instituto Politécnico Nacional, Mexico City, MEX; 3 Clinical Research Training Center, Centro Médico Nacional Siglo XXI, Mexico City, MEX; 4 Division Headquarters, Hospital de Gineco Obstetricia No. 3, Dr. Víctor Manuel Espinoza de los Reyes Sánchez, Centro Médico Nacional La Raza, Mexico City, MEX; 5 Biomedicine, Facultad de Medicina, Benemérita Universidad Autónoma de Puebla, Puebla, MEX

**Keywords:** combined nutrition, parenteral nutrition, vlbw, nicu, nutrition

## Abstract

Background

Adequate nutritional support is crucial for achieving optimal growth and development in very-low-birth-weight (VLBW) preterm infants. This study evaluated the efficacy of combined nutrition (CN) (parenteral plus enteral nutrition (EN)) as an alternative nutrition protocol for VLBW infants in the neonatal intensive care unit (NICU).

Methods

This retrospective cohort study collected clinical and growth data from the medical records of VLBW infants weighing between 1,000 and 1,500 grams in the NICU of the Hospital of Obstetrics and Gynecology "Dr. Víctor Manuel Espinosa de los Reyes Sánchez" of the Centro Médico Nacional "La Raza" Instituto Mexicano del Seguro Social, Mexico. Parenteral nutrition (PN) alone or CN (PN plus EN) was used for nutritional management. Statistical tests, such as Student's t-test, Mann-Whitney U test, and chi-square test as appropriate, were used to compare the clinical characteristics and growth data of the two groups, and relative risk was calculated to determine the probability of comorbidities according to feeding type. Statistical significance was set at p<0.05.

Results

The study included 90 VLBW infants, with 27 receiving PN alone and 63 receiving CN. No statistically significant differences were found concerning sex, age, or Apgar score. The CN group showed better weight gain with statistically significant differences at 28 days (p=0.002), with no increase in the relative risk of necrotizing enterocolitis (NEC) or other complications.

Conclusions

The CN protocol met the caloric and nutritional needs, without increasing morbidity and mortality. The protocol had a positive impact on weight gain and a shorter NICU stay and should be considered as a nutritional alternative for VLBW infants.

## Introduction

Very-low-birth-weight (VLBW) preterm infants (less than 1,500 grams) are subjected to physiologic and metabolic stress that increases their nutrient requirements. Their gastrointestinal immaturity provides insufficient energy and nutrients to meet their increased requirements, with their limited nutrient reserves rarely achieving optimal postnatal growth; therefore, most VLBW infants accumulate nutrient deficits during their hospital stay [1].

Several guidelines have been established in nutritional management to maintain higher homeostasis requirements [2-4]. Evidence on nutrition with human breast milk on VLBW infants during hospitalization in neonatal intensive care units (NICUs) shows a decreased risk of developing infections and necrotizing enterocolitis (NEC) [5,6]. Mother’s own milk is the recommended form for enteral nutrition (EN) for VLBW infants given that it reduces the probability of severe comorbidities; nevertheless, these infants require higher amounts of nutrients, which cannot be attained merely from breast milk. In addition, some mothers cannot produce and sustain enough milk to meet their nutritional needs [7,8].

Donor human milk (HM) should be the second choice, i.e., the milk comes from donors who are selected after health screening, which must be appropriately pasteurized and managed [9,10]. However, protein content is often inadequate, resulting in poor growth. The provision of donor milk is common in some hospital NICUs in developed countries. However, there is no global coordination of HM banks, which should establish minimum quality, safety, and ethical standards to guide national policies. Therefore, HM banks must adapt to the various restrictions, resources, and needs present in their local areas worldwide, where different legal statuses, unavailability, and non-standardized practices may exist [11,12].

As a result, parenteral nutrition (PN) is critical for the care of VLBW infants as it provides a relatively safe means of meeting nutrient intakes. PN is a highly complex combination of amino acids, lipid emulsions, carbohydrates, electrolytes, vitamins, and minerals that differs significantly from adult PN. To date, there is no international consensus regarding the ideal growth pattern for preterm VLBW infants [3,13,14].

The introduction of enteral feeds for VLBW infants is often delayed because it may not be well tolerated or increase the risk of NEC, but enteral feeds may have improvements by increasing nutrient intake and growth rates; accelerating intestinal physiological, metabolic, and microbiomic postnatal transition; and reducing the risk of complications associated with intravascular devices for fluid administration [2,5].

The growth delay and complications in VLBW infants have been attributed to inadequate nutrient intake and lack of standardization in feeding practices. Consequently, evaluating new feeding practices in VLBW infants is needed, especially when local conditions are questionable. Hence, the purpose of the present study was to evaluate combined nutrition (CN) (PN plus EN by an orogastric tube) in the increment of weight gain and complications in a Mexican hospital NICU.

## Materials and methods

Study design and patients

A retrospective cohort study was conducted at the NICU of the Hospital of Obstetrics and Gynecology "Dr. Víctor Manuel Espinosa de los Reyes Sánchez" of the Centro Médico Nacional "La Raza" Instituto Mexicano del Seguro Social, Mexico. After obtaining authorization of the institutional ethics and research committees (R-2015-3702-43), clinical and growth data were collected from the files of premature VLBW infants born during 2020. The inclusion criteria were infants with gestational age <37 weeks, weighing between 1,000 and 1,500 grams, with Apgar scores >4, admitted to the NICU immediately after birth up to 72 hours, with a minimum NICU stay of 28 days, and with complete clinical data in the medical history. Infants with congenital metabolic diseases and severe malformations, such as gastroschisis and complex cardiopathies precluding formula feeding or HM, were excluded from the study. Infants who died during hospitalization were excluded. No chromosomopathies were detected in the study population.

Nutritional management

PN was started within the 48 hours to 72 hours of life. The PN solution containing amino acids (compound amino acid crystal injection 8%), lipids (20% medium- and long-chain fat emulsion injection), dextrose 50%, minerals, trace elements, water-soluble vitamins, vitamin K, and heparin was administered daily and infused continuously for 24 hours. The caloric intake by PN was 140-180 calories per day.

CN was initiated within the first 72 hours of life, involved the administration of a combination of PN along with EN, preterm formula (PreNan, Nestle, Germany), delivered every three hours through an orogastric tube placed for 12 hours. Oral intake comprises 80-110 calories plus 60-80 calories provided parenterally, resulting in a combined total of 160-180 calories per day, which is comparable to the parenteral supply.

Data analysis

Data were expressed as group means ± standard deviation (SD), as the median (interquartile range (IQR) 25. 75), or as a number (percentage), as appropriate. The Kolmogorov-Smirnov test was used to corroborate the normality of the quantitative variable weight gain. The type of food received and the weight in grams were determined on days 14, 21, and 28. The clinical characteristics of the two groups were compared using Student's t-test, Mann-Whitney test, or chi-square test as appropriate. Relative risk was calculated to determine the probability of comorbidities according to the feeding type. Statistical analyses were performed using IBM SPSS Statistics for Windows, Version 23 (released 2015; IBM Corp., Armonk, New York, United States). Statistical significance was set at p<0.05.

## Results

**Figure 1:**
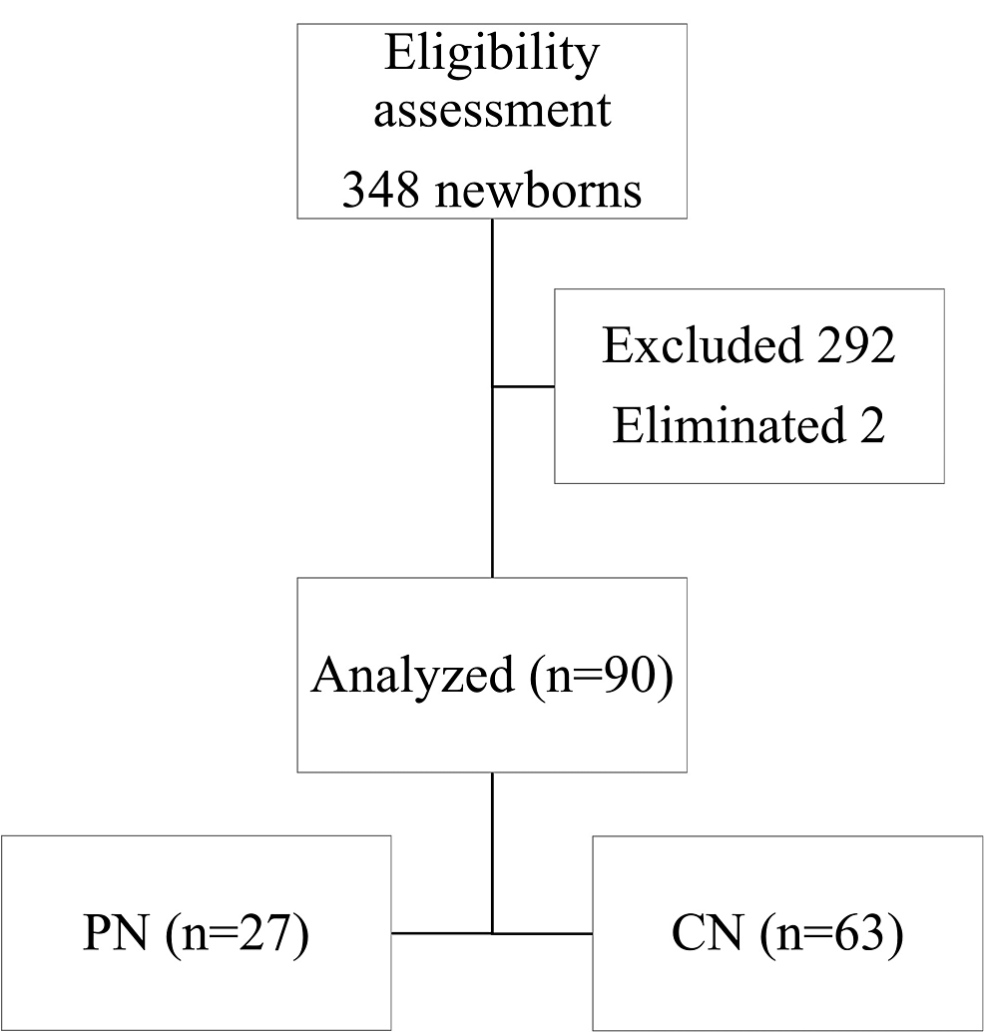
Flowchart of eligibility assessment of all preterm infants hospitalized in the neonatal intensive care unit during the study period.

From January to December 2020, all the records of 384 consecutively admitted preterm infants in the NICU were screened for this study. Among them, 292 patients were excluded due to failure to meet the inclusion criteria, and two patients who died in the first week of life were also excluded. The study included 90 VLBW preterm infants, with 27 (30%) receiving PN and 63 (70%) receiving CN (Figure 1).

Clinical characteristics, including sex distribution, Apgar scores, and gestational age, are summarized in Table 1. Apgar scores greater than 7 at five minutes were found in 85.71% of newborns in the CN group compared to 66.7% in the PN group. The gestational age of patients in the PN group ranged from 27-30 weeks (40.7%) to 31-33 weeks (59%), whereas most patients in the CN group had a gestational age of 31-33 weeks (73%). Nearly all patients (94.4%) received mechanical ventilation and 80% received prenatal steroids as a part of their management in the ICU. Preeclampsia was the predominant pathology in the mothers of these patients.

**Table 1:** Demographic characteristics of newborns weighing 1,000 to 1500 grams hospitalized in the neonatal intensive care unit. * p < 0.05 chi-square test

	Parenteral nutrition n (%)	Combined nutrition n (%)	p-value*
n	27 (30)	63 (70)	
Gender			
Male	15 (55.6)	27 (42.9)	0.26
Female	12 (44,.4)	36 (57.1)	
Apgar >7 at minute 5	18 (66.7)	55 (87.3)	0.02
Gestational age n (%)			
27-30 weeks	16 (59.2)	32 (50.7)	0.14
31-33 weeks	11 (40.8)	31 (49.3)	
Mechanical ventilation n (%)	27 (100)	58 (92.1)	0.13
Antenatal steroids n (%)	18 (60)	61 (92.4)	0.03*
Gestational diabetes n (%)	2 (10.6)	55 (83.3)	0.59
Preeclampsia n (%)	11 (34.8)	7 (7.7)	0.70

**Figure 2:**
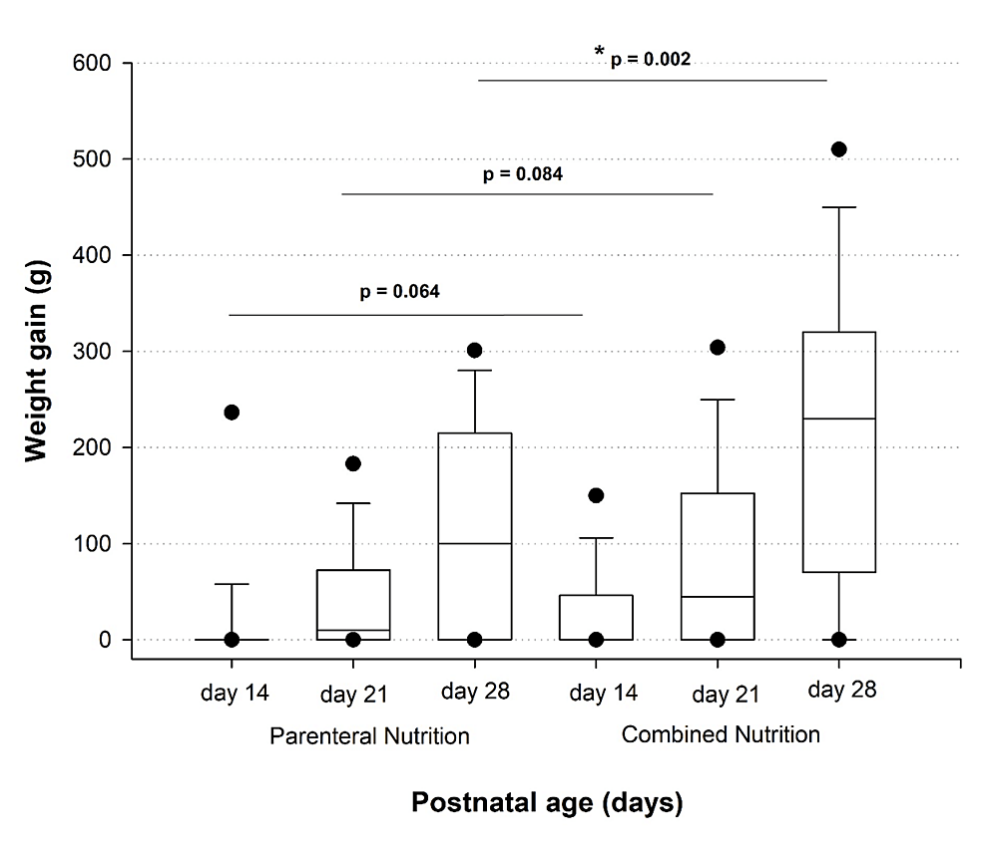
Changes in weight gain in very-low-birth-weight preterm infants at days 14, 21, and 28 with parenteral and combined nutrition. Data presented as median (interquartile range (IQR) 25. 75). * p=0.002 Mann-Whitney U test

Weight gain was found to be significantly higher in patients receiving CN, with more than 10 grams per day of weight gain observed in 23 newborns, while only four fed with PN demonstrated said gain (Figure 2). Differences in weight gain between the two groups were statistically significant at 28 days (p=0.002) and showed trends toward significance at 14 days (p=0.064) and 21 days (p=0.084).

Finally, more than 50% of the patients in both groups developed retinopathy of prematurity (ROP). Bronchopulmonary dysplasia (BPD) was also common in both groups, affecting 74.1% of patients in the PN group and 66.7% in the CN group, with a relative risk of 1.11 (95% confidence interval: 0.83-1.47). The incidence of enterocolitis, renal failure, and major intraventricular hemorrhage grade III was similar between the two groups, with relative risks of two for all the three complications, but none of these differences were statistically significant (Table 2).

**Table 2:** Complications of combined and parenteral nutritional therapy in the neonatal intensive care unit. RR: relative risk; CI: confidence interval

	Parenteral nutrition n (%)	Combined nutrition n (%)	RR	CI 95%
n	27 (30)	63 (70)		
Acute renal failure	3 (11.1)	3 (4.8)	2.33	0.50-10.83
Intraventricular hemorrhage grade III	5 (18.5)	5 (7.9)	2.33	0.73-7.40
Retinopathy of prematurity	16 (59.3)	32 (50.8)	1.16	0.78-1.73
Bronchopulmonary dysplasia	20 (74.1)	42 (66.7)	1.11	0.83-1.47

## Discussion

Multiple factors impact the survival of VLBW preterm infants in the NICU, including continuous monitoring, the presence of surfactants, mechanical ventilation, and effective nutrition management. The prolonged NICU stay exposes them to poor feeding and insufficient intake of necessary essential nutrients, so they often do not gain weight according to expectations causing postnatal growth failure, which is associated with significant neurodevelopmental impairments [15,16]. Both postnatal growth and quality of early nutritional support have been recognized as capital factors contributing to better evolution in hospital NICUs [17].

The establishment of quality-defined nutritional care protocols can improve weight gain in VLBW preterm infants [18]. The current study provides important findings on the weight gain of VLBW infants receiving CN (EN plus PN) contrasted with those receiving only PN, indicating that the characteristics and requirements of VLBW infants are greater due to their constitution, gestational age, and associated pathology such as respiratory distress syndrome, hydroelectrolytic and metabolic alterations, infectious processes, BPD, and ROP.

Designed early, fully balanced PN is vital to provide adequate and balanced energy, macronutrients, and micronutrients to support growth and prevent deficiencies in preterm VLBW infants after delivery [19,20]. Even though parenteral feeding implies the individualization of the patient and his treatment, knowing the specific disorder he suffers from and his feeding difficulties, in this study and as the evidence showed, the use of standardized PN is a valid alternative to individualized PN [19,21]. Early initiation of PN ensures that the newborn survives critical disease states, and the use of EN plus PN initiates the functioning of the microbiota by arresting the process of catabolism.

PN should not be used as a long-term substitute for EN [22]. Debate persists on the care of VLBW infants regarding the type, initiation, and advancement of EN that maintains infant growth. In VLBW infants, early total enteral feeding is tolerated in the first postnatal period (even within the first 14 hours) with no associated increase in NEC or death [23,24]. In this study, PN was employed within the first 24 hours, and in CN formula, enteral feeding with an orogastric tube was started between 24 hours and 72 hours with a 28-day follow-up, which produced an improvement in growth without an increase in the relative risk of NEC and other complications (Table 2). Proper nutrition guidelines should be established for an adequate start of feeding in sufficient quantity, at adequate concentrations, and on an individual basis.

Increased use of combined or exclusive EN therapy is required for patients who can tolerate it, with an adequate starting regimen of HM, even when combined with PN. This approach offers the greatest benefits for growth and development in the NICU. Preterm infants have insufficient nutrient reserves at birth; therefore, the use of the mother's colostrum or supplementation of HM with the required nutrients is recommended for all preterm infants [25]. Observational data suggest a reduced risk of NEC among VLBW infants fed with breast milk [26]. Initiation of HM feeds on the first postnatal day has been associated with achieving full feeds, reducing the need for central lines and PN, and promoting higher growth rate [27]. Omitting residual gastric assessment increases EN delivery and improves weight gain [28]. Finally, a more rapid advancement of feeding volumes has been associated with a shorter duration to achieve full feeds [29].

The literature is consistent about the advantages of early enteral feeding, especially with the use of HM [30], but not all mothers can provide their child with enough milk, and pasteurized human donor milk from an established milk bank is not always available; alternatives are required [9]. PN is considered an alternative for patients with strict fasting, and the transition to EN should be made as soon as possible. CN in this study showed greater benefits than exclusive PN. Currently, there are no reviews about combining nutrition in VLBW premature infant feeding.

The limitations of the study include the fact that it was based on the collection of data from clinical records. Therefore, we were not able to perform exact daily calculations of calorie and fluid intake or measure lean or fat reserves, which could differentiate weight gain from edema. In addition, because patients stayed in the NICU during the COVID-19 pandemic, HM was not used at any time, and only formula and PN were given for a minimum duration of 14 to 45 days to establish the diagnosis of ROP or BPD. We selected patients weighing between 1,000 and 1,500 grams because this population received constant enteral intake during the first 14 days of life. However, those under that weight often fasted for more than 14 days and showed distinct and often deadly progressive evolution. As this was a retrospective observational study, we could not control for these variables. Nevertheless, we emphasize that, in the absence of HM, formula milk plus PN seems to be useful for gaining weight in critically ill premature infants.

## Conclusions

The present study demonstrated that the CN protocol met caloric and nutritional needs without increasing morbidity and mortality. In addition, it had a positive impact on weight gain. Therefore, this protocol should be considered as an alternative for feeding VLBW infants. Decision-making is extremely important when feeding preterm infants to NICUs. Further studies are needed to establish the best types and methods of nutrient administration in critically ill patients based on the specific pathology. Much remains to be learned about nutrient administration in VLBW preterm infants.
